# The influence of different negative feedback on the decay of self-deception

**DOI:** 10.3389/fpsyg.2024.1499089

**Published:** 2025-01-23

**Authors:** Juan Liu, Wenjun Ding, Liying Deng, Min Tan, Peipei Guan

**Affiliations:** ^1^School of Education Science, Hunan Normal University, Changsha, China; ^2^Cognition and Human Behavior Key Laboratory of Hunan Province, Changsha, China; ^3^Key Laboratory for Big Data of Basic Education, Hunan Normal University, Changsha, China; ^4^School of Design, Guangxi Normal University, Guilin, China; ^5^King’s College London, London, United Kingdom

**Keywords:** self-deception, negative feedback, decay, positive beliefs, cheating

## Abstract

**Introduction:**

Though some studies have found the positive influences of moderate self-deception on individuals and society, there are many that have shown its negative influences on individuals and society. Long-term self-deception will have negative influences which could cause high individual losses and even social disasters. Therefore, it is essential to abate the decay of self-deception to avoid its negative influences and help individuals to better monitor themselves.

**Methods:**

In this research, we explored the impact of various types of negative feedback on the decay of self-deception using a forward-looking paradigm with three conditions: no-feedback, ambiguous negative feedback, and real negative feedback. The experiment under each condition was tested four times. The negative feedback was provided after Tests 2 and 3.

**Results:**

The results indicated that, in Test 1 of both Experiments 1 and 2, the answer group demonstrated notably stronger positive beliefs and a higher propensity for cheating compared to the control group. Additionally, self-deception was more pronounced under the no-feedback than under the negative feedback in the subsequent three tests. Furthermore, the condition of ambiguous negative feedback led to greater self-deception in the final three tests compared to the condition of real negative feedback in Experiment 2. The results also revealed that self-deception gradually diminished with real feedback in the answer group.

**Discussion:**

The findings showed that both ambiguous and real negative feedback reduce self-deception, although real negative feedback having a greater effect than ambiguous feedback. Additionally, the reduction of self-deception was fundamentally related to a decrease in unrealistic positive beliefs, and this decline in self-deception was also influenced by monetary rewards.

## Introduction

Self-deception is understood as a persistent positive belief about oneself, which endures despite contradictory evidence (Chance et al., 2011; van der Leer and McKay, 2017). It is also considered a form of positive bias, where individuals unconsciously sustain favorable perceptions of themselves (Hildebrand et al., 2018). Such bias occurs at various stages of information processing. People will selectively extract, mask information, or reconstruct memories to search and attend to their expected information rather than real information (Von Hippel and Trivers, 2011). Promoting adaptive value and regulate individuals’ mental health, subjective wellbeing, and interpersonal relationship are positive influences of moderate self-deception (Lopez and Fuxjager, 2012; Seiffert-Brockmann and Thummes, 2017; Sheridan et al., 2015; Butterworth et al., 2022). Self-deception makes individuals unable to clearly understand themselves, which will hinder their long-term development (Kaczmarek and Gaś, 2021). For example, denying sickness leads to impaired health (Sims, 2017), negatively impacted the acquisition of knowledge (Junbang et al., 2023). The conflict of individual between ethical standard and self-interest is regulated by self-deception (Batson et al., 1999; Tang et al., 2018; Tang et al., 2017). Self-deception as a strategy and moral failure (MacKenzie, 2022), it can make an individual’s immoral behavior be mistaken as moral and promote unethical and cheating behavior. Some men believe that violence against their partners is moral (Vecina, 2018; Vecina et al., 2016). Self-deception is widespread in organizations and impedes the process of critical reflection (Kuntz and Dehlin, 2019). It even leads to the bankruptcy of enterprises (Chance et al., 2015), the generation of government corruption (Desai et al., 2018) and the destruction of global cooperative behaviors (Babino et al., 2018). Chance et al. (2011) found that when using money rewards to encourage participants to accurately predict their performance, they would still over predict their future performance, suggesting that self-deception might come at a high price. The positive influence of self-deception is a short-term instant reward, but long-term self-deception will have negative influences. The decay of self-deception can not only help individuals to better monitor themselves, but also lessen the detrimental impact of self-deception within society.

Currently, there is limited research on the decline of self-deception. Individuals are more likely to utilize social strategies of self-deception to more effectively deceive others (Butterworth et al., 2022; Liu et al., 2019). Prior research had indicated that inducing cheating could bolster one’s positive beliefs, which in turn promoted self-deception. In other words, there is a reciprocal and interdependent relationship among cheating, positive beliefs, and self-deception. Chance et al. (2015) examined this phenomenon by giving participants real performance feedback following the second and third tests in a sequence of four consecutive assessments. The results show that self-deception gradually decayed with the repetition of real performance feedback. In addition, the dot-tracking task of the fast group in real performance feedback condition had slower speed than the ambiguous performance feedback in the testing phase (Sloman et al., 2010). Therefore, feedback is a method to promote the decay of self-deception. Feedback is described as information given to learners by others from the outside world, such as result, performance, or achievement. The purpose of such feedback is to influence learners’ motivation, cognition and behavior to enhance their performance. When individuals are given feedback, the valence of negative feedback is a factor that cannot be ignored. Negative feedback involves a critical evaluation by an assessor of another person’s products, performance, or characteristics (Duijnhouwer et al., 2012). The theory of belief adjustment refers to the process by which a rational agent transitions their beliefs from one state to another. It is an important component of human intellectual activity. When faced with inconsistent information, individuals do not completely modify their original beliefs; rather, they tend to readjust the intensity of those beliefs (Johnson-Laird et al., 2004). According to belief adjustment theory, self-deception is a positive belief that can be adjusted by inconsistent belief. That is, negative belief may adjust and decay self-deception. As negative beliefs primarily stem from external feedback, they serve as an effective means to reduce self-deception. Liu et al. (2019) examined the influence of negative feedback on the positive beliefs associated with self-deception. Such work showed that the negative feedback which is provided before self-deception occurs impedes the establishment of positive beliefs and weakens an individual’s future positive beliefs, reducing self-deception. However, additional research is required to understand how negative feedback contributes to the reduction of self-deception.

Chance et al. (2015) confirmed real feedback can effectively reduce self-deception. Sloman et al. (2010) showed that ambiguous performance feedback is essential for the emergence of self-deception. Based on the above two studies, feedback can be divided into two kinds (ambiguous and real). People often get feedback from themselves or others’ evaluations involving their behavior in real life (Raftery and Bizer, 2009; Duijnhouwer et al., 2012). This feedback also included ambiguous and real feedback. For individuals, the real feedback that is not conducive to maintain positive beliefs about oneself is a kind of negative feedback for self-deceiver. Consequently, we divide negative feedback into ambiguous negative feedback and real negative feedback, and take no-feedback as the control condition. The present study aims to address the gap in previous experiments that focused only on ambiguous or real feedback, exploring how different types of negative feedback influence the decay of self-deception over time, further offering a fresh perspective for advancing research on the decay of self-deception, along with a deeper theoretical foundation and behavioral evidence for understanding this process.

Self-deception was evaluated through both inter-group and intra-group comparisons within a forward-looking paradigm. The inter-group comparisons revealed that the answer group’s predicted scores surpassed those of the control group (Chance et al., 2011). Intra-group comparisons examined self-deception by identifying instances where the predicted scores exceeded actual scores within the answer group (Chance et al., 2015). Inter-group comparisons provide a convenient method for evaluating self-deception between the two groups, while intra-group comparisons track changes in self-deception within the answer group. Cheating and Positive beliefs behaviors were measured by comparing the actual and estimated scores between the answer group and the control group.

Conclusively, this research extends the work of prior scholars by utilizing a forward-looking paradigm and the belief adjustment theory to examine the effects of different negative feedback on the decay of self-deception. It tackles unresolved issues from prior investigations, enriches the theoretical landscape of self-deception dissipation, and offers empirical behavioral validation for the dissipation process in individuals. This study examines the following questions. First, after individuals establish positive belief, how repeated negative feedback impact the decline of self-deception over time? Second, what is the difference in the influence of repeated real and ambiguous negative feedback on the decay of self-deception over time? Third, what is the difference in the decay of self-deception as measured by inter and intra-group comparisons? In Experiment 1, the impact of repeated negative feedback on the decay of self-deception was examined. In Experiment 2, we investigated how different types of negative feedback (real and ambiguous) influenced the gradual decline of self-deception over time. In both experiments, inter-group and intra-group methods were used to assess self-deception and self-deception decay.

## Experiment 1

The objective of Experiment 1 was to investigate the impact of repeated negative feedback on the gradual reduction of self-deception over time. The study hypothesizes that the answer hints induce participants to establish positive beliefs, cheat in test, the occurrence of self-deception. It is also proposed that repeated exposure to negative feedback contributes to a gradual decrease in self-deception.

### Methods

#### Participants and design

The study’s experimental protocol received approval from the Ethics Committee of the Institute of Psychology at Hunan Normal University. We recruited 64 college students who were not majoring in psychology (56 males and eight females, with an average age of 19.45 ± 0.65 years) as participants. All participants were right-handed and had no previous experience in similar experiments. Participants were randomly allocated to the answer group (*n* = 34) or the control group (*n* = 30). Written informed consent was obtained from all participants, and they were compensated with 10 RMB upon completion of the experiment. Experiment 1 utilized a 2 (feedback type: no-feedback, negative feedback) × 2 (group: answer group, control group) mixed-design. The feedback type was used as a within-subject variable, and group as a between-subject variable. The dependent variables were positive beliefs, cheating, self-deception, and the decay of self-deception.

#### Materials

**Figure 1:**
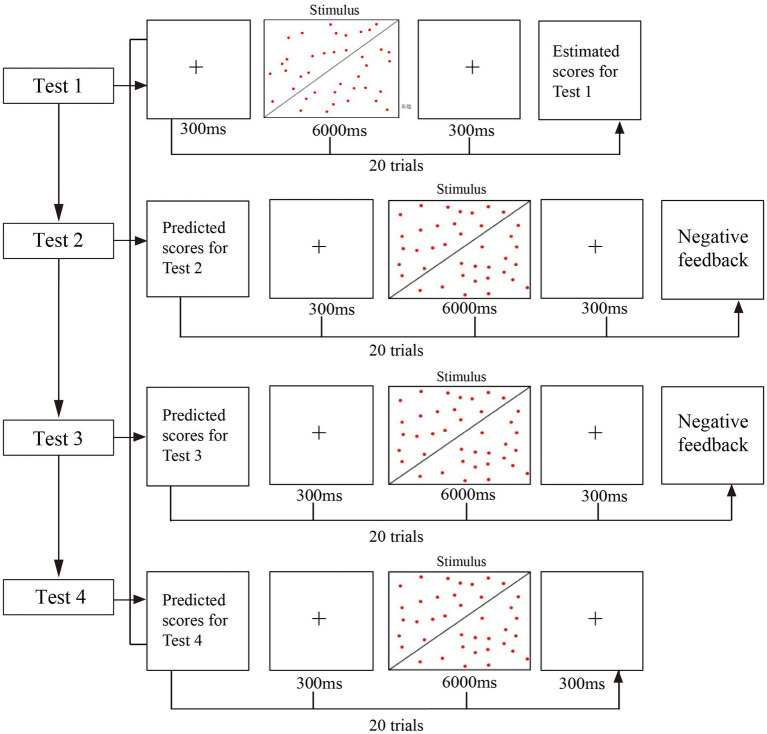
Experimental procedure of answer group.

The experimental materials consisted of 160 red dot images, also known as the dot estimation task (Liu et al., 2019; Fan et al., 2019; Fan et al., 2024; Fan et al., 2022). Each test included 20 red dot images, with each image containing either 39 or 40 red dots (see Figure 1). The set comprises seven graphs with more dots on the left side, seven graphs with a higher quantity of dots on the right side, and six graphs with an equal quantity of dots on both sides. The number of red dots on the left and right sides of each graph is nearly identical to increase the ambiguity of the responses, and the answer options are divided into three categories: left, right, and equal. It is difficult for participants to accurately estimate the number of points, and they have to choose between ambiguous judgments. Each graph takes the form of a trial, each of which takes 6 s. Participants intuitively judge which side has more or equal numbers of points. They press the “F” key when more points on the left, the “J” key when more points on the left, the “Y” key when equal number of dots.

### Procedure

Each participant was required to perform the test under no-feedback condition and negative feedback condition. After completing the test under no-feedback condition, participants chose the rest time independently and then carried out the test of negative feedback condition. The experimental process in negative feedback is shown in Figure 1. Each participant completed four tests under each condition. Upon finishing Test 1, participants were asked to evaluate their performance and forecast their scores for Test 2. The two negative feedbacks were provided after the second and third tests in four continuous tests. After the completion of Test 2, they received the first round of negative feedback and were required to approximate their scores for Test 3. After completing Test 3, participants were given a second round of negative feedback and asked to predict their scores for Test 4, which they completed afterward.

The only difference between the experimental procedure in the no-feedback and the above procedure is that no negative feedback was provided to participants. Liu et al. (2019) believed that no feedback is the highest type of ambiguous feedback, and “your score is low” is relatively accurate negative feedback. To improve the accuracy of the negative feedback, the negative feedback employed in this experiment was slightly adjusted from that used by Liu et al. (2019). The negative feedback provided to participants was “your score is lower than average.” Answer keys were provided below the images for the answer group, while participants in the control group did not receive any keys.

#### Assessment of positive beliefs, cheating, and self-deception

Positive beliefs were assessed by comparing the estimated scores of the two groups in Test 1, with higher estimates indicating stronger beliefs. Cheating was evaluated by comparing the actual scores of the two groups, where the answer group outperformed the control group in Test 1. Self-deception was measured through both inter-group and intra-group comparisons in Test 2. Under the impact of repeated negative feedback, the decay of self-deception was measured through both inter-group and intra-group comparisons across Tests 3 and 4. Inter-group comparisons focused on whether The answer group exhibited higher predicted scores compared to the control group, despite no significant differences in actual scores. Intra-group comparisons examined instances where predicted scores exceeded actual scores within the same group.

#### Data statistics and analysis

A multivariate analysis was conducted to evaluate the discrepancies between actual and estimated scores in Test 1, as well as the differences between actual and predicted scores in the subsequent three tests across both groups under different feedback conditions. The objective was to examine the effects of feedback on cheating behavior, positive beliefs, self-deception and the decay of self-deception. A repeated-measures ANOVA with a 2 (feedback type: no-feedback, negative feedback) × 2 (group type: answer group, control group) design was conducted to evaluate scores for Tests 3 and 4, aiming to identify self-deception and its reduction. A paired sample *t*-test was applied to analyze the difference between scores within the answer group, highlighting self-deception and its reduction through inter-group comparison. Since self-deception was not observed in the control group, its results were compared solely with those of the answer group.

### Results

#### Positive beliefs and cheating

**Figure 2:**
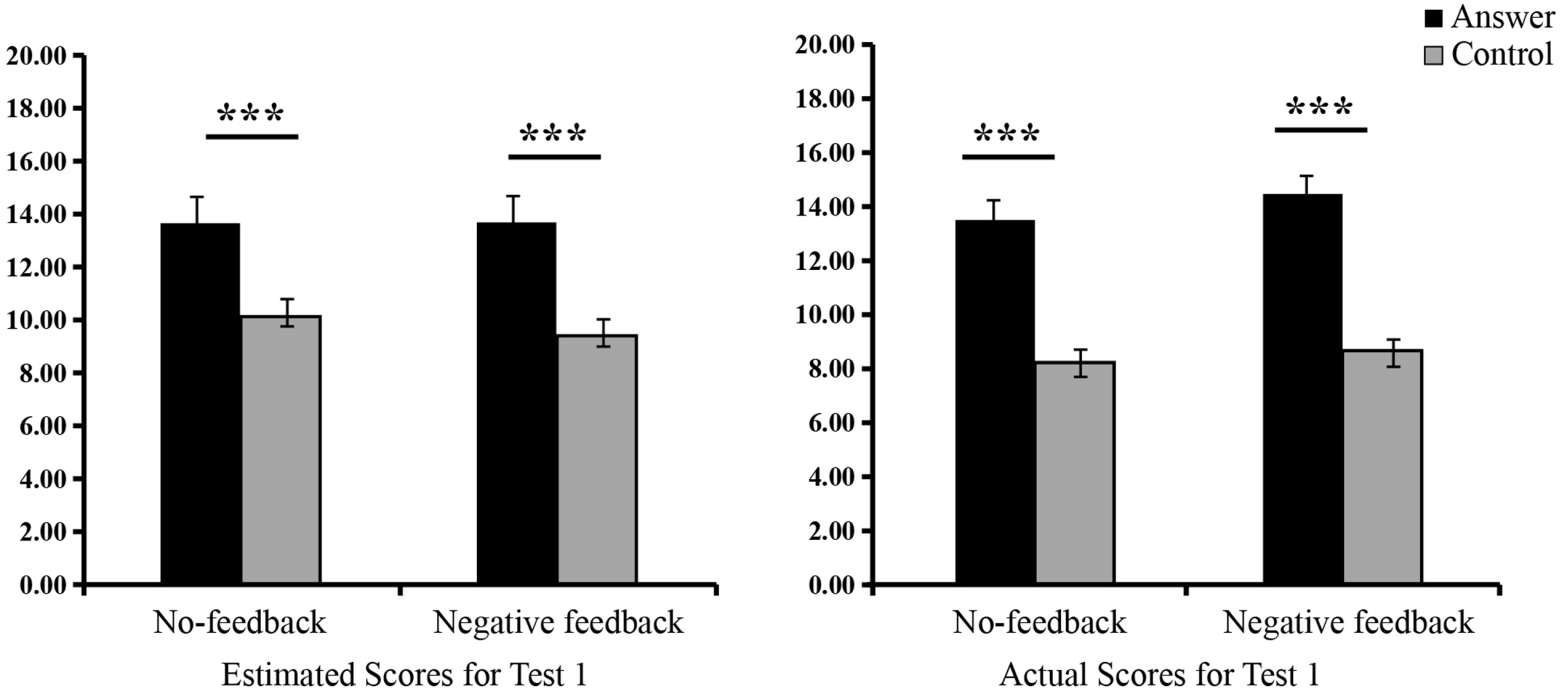
Scores for Test 1. ^∗^*p <* 0.05, ^∗∗^*p <* 0.01, ^∗∗∗^*p <* 0.001.

Analysis of variance revealed that under the no-feedback, the answer group had substantially higher actual and estimated scores compared to the control group [*F*_(1, 62)_ = 28.30, *p* < 0.001, $\eta_{p}^{2}$ = 0.31 and *F*_(1, 62)_ = 17.90, *p* < 0.001, $\eta_{p}^{2}$ = 0.22]. Similarly, in the negative feedback, substantial differences were observed between the answer and control groups in both actual and estimated scores [*F*_(1, 62)_ = 35.52, *p* < 0.001, $\eta_{p}^{2}$ = 0.36 and *F*_(1, 62)_ = 28.30, *p* < 0.001, $\eta_{p}^{2}$ = 0.31] (see Figure 2 and Table 1). These findings revealed that the answer group engaged in cheating by accessing the answers and subsequently developed positive beliefs about their performance under the no-feedback and negative feedback.

**Table 1:** The scores for Tests 1, 2, 3, and 4 under different experimental conditions (*M ± SD*).

			Test 1	Test 2	Test 3	Test 4
No-feedback	Answer group	Scores	13.65 ± 3.67	12.65 ± 2.47	11.18 ± 2.61	11.24 ± 2.23
		Actual scores	13.50 ± 5.03	7.15 ± 2.20	7.91 ± 2.11	8.32 ± 1.87
	Control group	Scores	10.13 ± 2.86	10.97 ± 2.46	10.43 ± 3.20	10.53 ± 3.30
		Actual scores	8.23 ± 2.22	7.43 ± 1.94	7.63 ± 1.92	7.57 ± 1.92
Negative feedback	Answer group	Scores	13.68 ± 3.33	11.44 ± 2.43	9.62 ± 2.22	8.97 ± 2.02
		Actual scores	14.47 ± 5.06	8.47 ± 2.02	7.71 ± 2.39	8.24 ± 1.88
	Control group	Scores	9.40 ± 3.07	10.10 ± 2.78	8.67 ± 2.40	8.13 ± 2.18
		Actual scores	8.67 ± 1.79	8.53 ± 1.18	7.30 ± 2.58	8.07 ± 2.24

#### Self-deception for Test 2 by inter-group comparison

**Figure 3:**
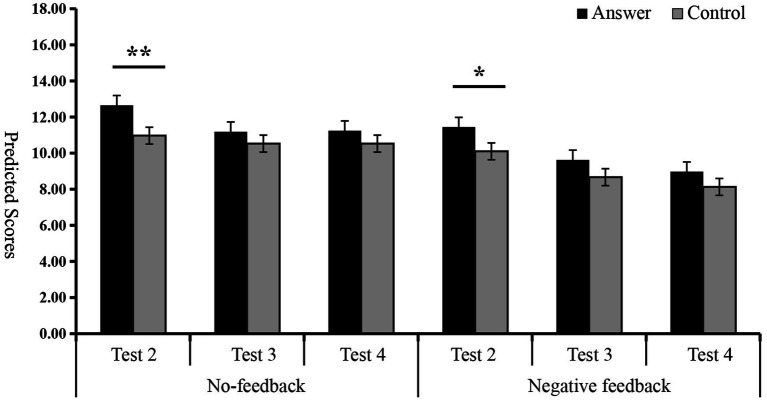
Inter-group comparison of predicted scores for Tests 2, 3, and 4. ^∗^*p <* 0.05, ^∗∗^*p <* 0.01, ^∗∗∗^*p <* 0.001.

The multivariate analysis revealed that in Test 2, the predicted scores of the control group under the no-feedback were significantly lower than those of the answer group [*F*_(1, 62)_ = 7.41, *p* < 0.01, $\eta_{p}^{2}$ = 0.11]. Under the negative feedback, the answer group also had significantly higher predicted scores in Test 2 compared to the control group [*F*_(1, 62)_ = 4.24, *p* < 0.05, $\eta_{p}^{2}$ = 0.06]. However, there were no noticeable differences in actual scores between the answer and control groups in either the no-feedback or negative feedback [*F*_(1, 62)_ = 0.30, *p* > 0.05, *F*_(1, 62)_ = 1.02, *p* > 0.05] (see Figure 3 and Table 1). These results indicate that the answer group engaged in self-deception under conditions of no-feedback and negative feedback.

#### Self-deception for Tests 3 and 4 by inter-group comparison

The repeated measures analysis of variance of the predicted scores indicated that there was no main effect of the group in Test 3 [*F*_(1, 62)_ = 2.16, *p* > 0.05]. The main effect of the group on actual scores was not observed [*F*_(1, 62)_ = 0.66, *p* > 0.05]. The main effect of feedback type in actual scores was not apparent [*F*_(1, 62)_ = 0.66, *p* > 0.05]. No interaction of group and feedback condition in both predicted and actual scores emerged [*F*_(1,62)_ = 0.34, *p* > 0.05, *F*_(1,62)_ = 0.03, *p* > 0.05]. However, a main effect of feedback type was found, with predicted scores significantly higher under the no-feedback condition than under the negative feedback [*F*_(1, 62)_ = 28.18, *p* < 0.001, $\eta_{p}^{2}$ = 0.31].

The two-factor repeated-measures analysis of variance revealed no clear main effect of the group on the predicted scores in Test 4 [*F*_(1, 62)_ = 2.20, *p* > 0.05]. A main effect of feedback type was found, showing that predicted scores were significantly higher under the no-feedback compared to the negative feedback [*F*_(1, 62)_ = 48.67, *p* < 0.001, $\eta_{p}^{2}$ = 0.44]. The main effect of group in actual scores was not apparent [*F*_(1, 62)_ = 0.29, *p* > 0.05]. The main effect of feedback type in actual scores was not apparent [*F*_(1, 62)_ = 2.20 *p* > 0.05]. The interaction between group and feedback did not significantly affect either the predicted or actual scores [*F*_(1, 62)_ = 0.04, *p* > 0.05, *F*_(1,62)_ = 0.59, *p* > 0.05].

To further examine the differences between the predicted scores of the answer and control groups under different feedback in Tests 3 and 4, multivariate analysis was used to analyze the predicted scores of the two groups in three tests, and to investigate the self-deception behavior and its decay. Under the no-feedback condition, the predicted scores for the answer and control groups in Tests 3 and 4 showed no noticeable difference [*F*_(1, 62)_ = 1.05, *p* > 0.05, *F*_(1, 62)_ = 1.12, *p* > 0.05]. Likewise, under the negative feedback, no significant difference was found between the predicted scores of the answer and control groups in Tests 3 and 4 [*F*_(1, 62)_ = 2.72, *p* > 0.05, *F*_(1, 62)_ = 2.54, *p* > 0.05] (see Figure 3 and Table 1).

The inter-group comparison results indicated no significant difference in predicted scores between the answer and control groups in Tests 3 and 4. This suggests that negative feedback eliminated self-deception in Tests 3 and 4. However, predicted scores under the no-feedback condition were significantly higher than those under the negative feedback condition, indicating that negative feedback accelerated the disappearance of self-deception.

#### Self-deception for Tests 2, 3, 4 by intra-group comparison

Intra-group comparisons examined self-deception by identifying instances where the predicted scores exceeded actual scores within the answer group (Chance et al., 2015), and tracked changes in self-deception within the answer group. Therefore, we only analyzed the performance of the answer group. The paired sample t-test was conducted to assess the relationship between feedback and scores. For the answer group, the predicted scores in Tests 2, 3, and 4 were substantially higher than the actual scores under the no-feedback: Test 2 [*t*(33) = 8.80, *p* < 0.001, *d* = 2.35], Test 3 [*t*(33) = 5.22, *p* < 0.001, *d* = 1.53], Test 4 [*t*(33) = 6.92, *p* < 0.001, *d* = 1.42]. In the negative feedback, the answer group showed higher predicted than actual scores for Tests 2 and 3: Test 2 [*t*(33) = 5.00, *p* < 0.001, *d* = 1.33], Test 3 [*t*(33) = 3.45, *p* = 0.002, *d* = 0.82], with no significant difference observed in Test 4 [*t*(33) = 1.49, *p* = 0.14, *d* = 0.37].

Intra-group analysis revealed that the predicted scores were substantially higher than the actual scores in Tests 2. The results of the intra-group comparison revealed that the predicted scores of the answer group in Test 3 were considerably higher than the actual scores, but this effect was no longer present in Test 4. These results indicate that revealed that self-deception was evident in Test 3 but disappeared by Test 4.

### Discussion

Test 1 was designed to investigate whether the answer hints induced establishment of cheating behavior, and self-positive beliefs of participants. The findings revealed that the answer group engaged in cheating by accessing the answers and subsequently developed positive beliefs about their performance. Test 2 was designed to examine the presence of self-deception. Inter-group and intra-group analysis results indicate that the answer group engaged in self-deception under conditions of no-feedback and negative feedback. These findings support our hypothesis and align with previous studies (Liu et al., 2019). Prior to Tests 3 and 4, negative feedback was administered to participants to study the impact of negative feedback on the decline of self-deceptive behaviors. Inter-group analysis results indicate that negative feedback influenced participants, reducing their positive beliefs about their test performance and leading to a decay in self-deception after the first instance of negative feedback in Test 3. However, intra-group comparison revealed that self-deception was evident in Test 3 but disappeared by Test 4. From the above results, it could be found that the observed self-deception decay time in Experiment 1 was different between the inter-group comparison and intra-group comparison. Therefore, we should ponder about what the reasons for these differences were. Self-deception is influenced by external motivations (Fan et al., 2017; Fan et al., 2022), such as money rewards (Chance et al., 2011; Chance and Norton, 2015). Experiment 1 might lack external motivation, so that the intensity of self-deception was not enough, and the phenomenon that self-deception was difficult to decay could not be observed in the inter-group comparison measured. Hence, how will the decay of self-deception change if greater positive beliefs are induced by increasing the motivation to cheat?

## Experiment 2

In Experiment 2, we expanded our investigation into the effects of varying types of negative feedback on the reduction of self-deception by introducing monetary incentives and distinguishing between ambiguous and real negative feedback. Hypothesis: (1) participants in the answer group under different feedback get influenced by answer hints to establish a positive belief and cheat for Test 1; (2) Participants in the answer group, compared to the control group, provided more accurate score predictions and exhibited self-deception during Test 2 across all three feedback; (3) Self-deception was higher in the ambiguous negative feedback than in the real negative feedback, but lower than in the no-feedback. Additionally, self-deception decreased at a slower rate under ambiguous negative feedback than under real negative feedback; (4) Predicted scores for Tests 3 and 4 varied depending on the type of negative feedback provided.

### Methods

#### Participants and design

The study’s experimental protocol received approval from the Ethics Committee of the Institute of Psychology at Hunan Normal University. Seventy-nine college students (55 males and 24 females, with an average age of 19.45 ± 0.65 years), who were not psychology majors, were recruited as participants. All participants were right-handed and had no previous experience in similar experiments. They were randomly assigned to either the answer group (*n* = 38) or the control group (*n* = 41). Informed written consent was obtained from all participants, and they were appropriately compensated upon completing the experiment. Experiment 2 employed a 3 (negative feedback type: no-feedback, ambiguous feedback, real feedback) × 2 (group: answer group, control group) mixed design. The negative feedback type used within-subject variables, while the group used between-subject variables. The dependent variables were positive beliefs, cheating, self-deception and the decay of self-deception.

#### Material

In this experiment, 240 red dot graphs were used, and each test included 20 red dot graphs. Others were the same as the dot estimation task materials used in Experiment 1.

#### Procedure

Participants were informed at the outset that their compensation would be determined by the accuracy of their responses, with 0.1 RMB awarded for each correct answer, up to a maximum of 24 RMB. Ambiguous was a relative concept, the negative feedback was relatively accurate feedback in relation to the no-feedback, while compared to the real negative feedback, the negative feedback in Experiment 1 was ambiguous negative feedback. The ambiguous negative feedback provided to participants was “your grade is lower than average” in Experiment 2 as in Experiment 1. The real negative feedback was to give participants the real scores of theirs, such as “the correct number of this test is 9.” The others were consistent with Experiment 1.

The evaluation of positive beliefs, cheating, and self-deception, as well as the data analysis methods used in Experiment 1, were also applied in Experiment 2.

### Results

#### Positive beliefs and cheating

**Figure 4:**
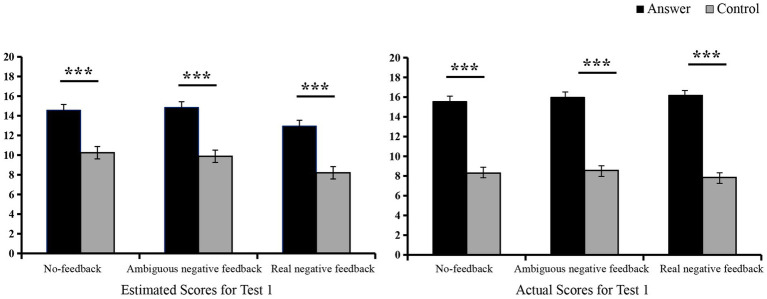
Scores in Test 1. ^∗^*p <* 0.05, ^∗∗^*p <* 0.01, ^∗∗∗^*p <* 0.001.

Analysis of variance showed that the answer group’s estimated scores were significantly higher than those of the control group across all conditions: no-feedback, ambiguous negative feedback, and real negative feedback [*F*_(1, 77)_ = 42.65, *p* < 0.001, $\eta_{p}^{2}$ = 0.59, *F*_(1, 77)_ = 76.53, *p* < 0.001, $\eta_{p}^{2}$ = 0.50, *F*_(1, 77)_ = 43.95, *p* < 0.001, $\eta_{p}^{2}$ = 0.36]. The difference between the actual scores of the experimental group and the control group was significant under the conditions of no-feedback, ambiguous negative feedback, and real negative feedback [*F*_(1, 77)_ = 80.15, *p* < 0.001, $\eta_{p}^{2}$ = 0.51, *F*_(1, 77)_ = 29.15, *p* < 0.001, $\eta_{p}^{2}$ = 0.28, *F*_(1, 77)_ = 96.43, *p* < 0.001, $\eta_{p}^{2}$ = 0.56] (see Figure 4 and Table 2). These findings revealed that the answer group engaged in cheating by accessing the answers and subsequently developed positive beliefs about their performance across all conditions.

**Table 2:** The scores for Tests 1, 2, 3, and 4 under different experimental conditions (*M ± SD*).

			Test 1	Test 2	Test 3	Test 4
No- feedback	Answer group	Scores	14.56 ± 3.71	13.76 ± 2.79	12.47 ± 3.07	12.21 ± 3.05
		Actual scores	15.55 ± 4.86	8.32 ± 2.04	7.66 ± 1.94	7.82 ± 2.04
	Control group	Scores	10.24 ± 1.93	11.76 ± 2.33	11.00 ± 2.67	10.98 ± 2.12
		Actual scores	8.29 ± 2.06	7.48 ± 2.16	7.56 ± 2.08	7.80 ± 2.11
Ambiguous negative feedback	Answer group	Scores	14.84 ± 3.97	12.97 ± 3.14	10.37 ± 2.30	10.02 ± 3.56
		Actual scores	15.97 ± 4.76	8.89 ± 2.22	8.65 ± 1.92	8.42 ± 2.36
	Control group	Scores	9.88 ± 2.59	10.76 ± 2.24	8.93 ± 2.44	8.15 ± 2.79
		Actual scores	8.56 ± 2.25	8.76 ± 1.73	8.20 ± 1.83	8.31 ± 1.57
Real negative feedback	Answer group	Scores	12.95 ± 4.93	10.39 ± 3.64	8.97 ± 3.16	8.34 ± 2.64
		Actual scores	16.18 ± 4.99	7.26 ± 1.64	7.92 ± 1.49	8.55 ± 2.39
	Control group	Scores	8.20 ± 2.63	8.34 ± 2.69	8.61 ± 2.27	8.60 ± 1.95
		Actual scores	7.85 ± 2.08	7.76 ± 2.36	7.88 ± 2.37	8.78 ± 1.73

#### Self-deception for Test 2 by inter-group comparison

**Figure 5:**
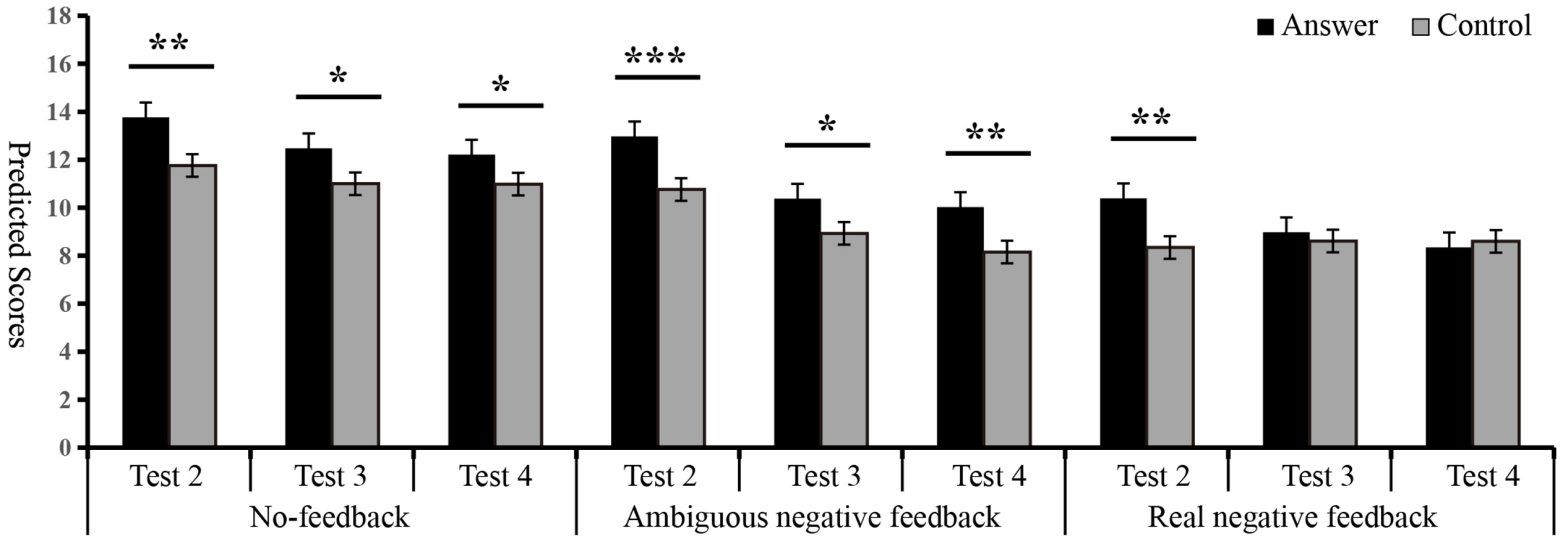
Inter group comparison of predicted scores for Tests 2, 3, and 4. ^∗^*p <* 0.05, ^∗∗^*p <* 0.01, ^∗∗∗^*p <* 0.001.

Multivariate analysis showed that the control group’s predicted scores were significantly lower than those of the answer group under all conditions: no-feedback, ambiguous negative feedback, and real negative feedback [*F*_(1, 77)_ = 13.45, *p* < 0.001, $\eta_{p}^{2}$ = 0.15, *F*_(1, 77)_ = 14.54, *p* < 0.001, $\eta_{p}^{2}$ = 0.16, *F*_(1, 77)_ = 9.50, *p* < 0.01, $\eta_{p}^{2}$ = 0.11] (see Figure 5 and Table 2). No significant difference was observed between the actual scores of the answer group and the control group under these conditions [*F*_(1, 77)_ = 3.6, *p* > 0.05, *F*_(1, 77)_ = 0.10, *p* > 0.05, *F*_(1, 77)_ = 1.14, *p* > 0.05]. These results indicate that the answer group engaged in self-deception under all conditions.

#### Self-deception for Test 3 by inter-group comparison

The repeated measures analysis of variance of predicted scores revealed that the main effect of the group was observed [*F*_(1, 77)_ = 5.51, *p* < 0.05, $\eta_{p}^{2}$ = 0.07]. The main effect of negative feedback type was observed [*F*_(2, 77)_ = 35.15, *p* < 0.001, $\eta_{p}^{2}$ = 0.31]. Moreover, predicted scores were significantly higher under the no-feedback compared to both the ambiguous (*p* < 0.001) and real negative feedback (*p* < 0.001). The predicted scores under the ambiguousr negative feedback were also significantly higher than those under the real negative feedback (*p* < 0.05). No significant interaction between group and type of negative feedback was found [*F*_(2, 77)_ = 1.53, *p* > 0.05].

The repeated measures analysis of variance of actual scores indicated that no main effect of the group [*F*_(1, 77)_ = 0.63, *p* > 0.05]. The main effect of negative feedback type was observed [*F*_(2, 77)_ = 3.49, *p* < 0.05, $\eta_{p}^{2}$ = 0.04]. The actual scores under the ambiguous negative feedback were significantly higher than those under the no-feedback (*p* < 0.05). However, no significant difference was found between the actual scores under the no-feedback and ambiguous feedback compared to those under the real negative feedback (p > 0.05). The interaction between group and negative feedback type emerged [*F*_(2, 77)_ = 0.27, *p* > 0.05].

In addition, the multivariate analysis was used to analyze the group differences in predicted scores under different feedback conditions to further investigate the self-deception behavior. There were apparent differences between the predicted scores of the answer and control group under the no-feedback and ambiguous negative feedback [*F*_(1, 77)_ = 5.19, *p* < 0.05, $\eta_{p}^{2}$ = 0.06, *F*_(1, 77)_ = 5.52, *p* < 0.05, $\eta_{p}^{2}$ = 0.07]. No significant differences were observed between the predicted scores of the answer group and the control group under the real negative feedback [*F*_(1, 77)_ = 0.35, *p* > 0.05] (see Figure 5 and Table 2).

These results suggest that the answer group exhibited self-deception the no-feedback and ambiguous negative feedback, while self-deception was not observed with real negative feedback. The findings also indicate that ambiguous negative feedback can effectively diminish self-deception, whereas real negative feedback is more effective in accelerating its decline.

#### Self-deception for Test 4 by inter-group comparison

The repeated measures analysis of variance of predicted scores indicated that the main effect of the group was observed [*F*_(1, 77)_ = 4.03, *p* < 0.05, $\eta_{p}^{2}$ = 0.05]. The main effect of feedback type was observed [*F*_(2, 77)_ = 47.68, *p* < 0.001, $\eta_{p}^{2}$ = 0.38], the predicted scores in the no-feedback were significantly higher than those in both the ambiguous (*p* < 0.001) and real negative feedback (*p* < 0.001). An apparent interaction between group and feedback type was observed [*F*_(1, 77)_ = 5.31, *p* < 0.01, $\eta_{p}^{2}$ = 0.06]. The answer group’s predicted scores in the no-feedback condition were significantly greater than those in both the ambiguous (*p* < 0.001) and real negative feedback (*p* < 0.001). An apparent difference between the predicted scores under the ambiguous and real negative feedback were observed (*p* < 0.01). The control group’s predicted scores in the no-feedback were significantly higher than those in both the ambiguous (*p* < 0.001) and real negative feedback (*p* < 0.001).

The repeated measures analysis of variance of actual scores indicated that no main effect of the group was observed [*F*_(1, 77)_ = 0.02, *p* > 0.05]. The main effect of feedback type was apparently [*F*_(2, 77)_ = 3.51, *p* < 0.05, $\eta_{p}^{2}$ = 0.04]. The actual scores in the real negative feedback were significantly higher than those in the no-feedback (*p* < 0.05), while there was no significant difference between the scores under the actual and ambiguous negative feedback (*p* > 0.05). There also was no apparent difference between the actual scores under conditions of the ambiguous negative feedback and no-feedback (*p* > 0.05). No significant interaction was found between the group and feedback type [*F*_(2, 77)_ = 0.14, *p* > 0.05].

In addition, another multivariate analysis was used for the group differences in predicted scores under different feedback conditions to further investigate the self-deception behavior. Apparently, differences between the predicted scores of the answer and control groups under the no-feedback and ambiguous negative feedback were observed [*F*_(1, 77)_ = 5.51, *p* < 0.05, $\eta_{p}^{2}$ = 0.07, *F*_(1, 77)_ = 5.94, *p* < 0.05, $\eta_{p}^{2}$ = 0.07], no significant difference was observed between the predicted scores of the answer and control groups under the real negative feedback [*F*_(1, 77)_ = 1.71, *p* > 0.05] (see Figure 5 and Table 2).

These results suggest that the answer group exhibited self-deception under the no-feedback and ambiguous negative feedback, while self-deception was not observed with real negative feedback. The findings also indicate that ambiguous negative feedback can effectively diminish self-deception, whereas real negative feedback is more effective in accelerating its decline.

#### Self-deception for Tests 2, 3, and 4 by intra-group comparison

The results for the answer group showed that predicted scores for Tests 2, 3, and 4 were markedly higher than the actual scores under the no-feedback, as indicated by the paired sample t-test: Test 2 [*t*(37) = 10.57, *p* < 0.001, *d* = 2.35], Test 3 [t(37) = 8.33, *p* < 0.001, *d* = 1.53], and Test 4 [*t*(37) = 6.50, *p* < 0.001, *d* = 1.42]. Under the ambiguous negative feedback, predicted scores for Tests 2, 3, and 4 also exceeded the actual scores: Test 2 [*t*(37) = 7.06, *p* < 0.001, *d* = 1.33], Test 3 [*t*(37) = 2.75, *p* = 0.009, *d* = 0.82], and Test 4 [*t*(37) = 2.13, *p* = 0.04, *d* = 1.42]. In the real negative feedback, the predicted score for Test 2 was significantly higher than the actual score [*t*(37) = 5.31, *p* < 0.001, *d* = 2.35], while no significant difference emerged between predicted and actual scores for Tests 3 and 4: Test 3 [*t*(37) = 1.90, *p* = 0.07, *d* = 1.53], and Test 4 [*t*(37) = 0.37, *p* = 0.71, *d* = 0.35].

Intra-group comparison results suggest that the answer group exhibited self-deception under the no-feedback and ambiguous negative feedback in Tests 2, 3, and 4. Under the real negative feedback, self-deception was observed in Test 2 but was notably absent in Tests 3 and 4.

### Discussion

Test 1 was designed to examine whether answer hints induced establishment of positive beliefs and cheating behavior. The results of Test 1 indicated the answer group engaged in cheating and developed positive beliefs about their performance. Test 2 was employed to investigate the presence of self-deception. Inter-group and intra-group comparisons confirming the occurrence of self-deception and aligning with both our hypothesis, the findings from Experiment 1 and align with previous studies (Liu et al., 2019). Before Tests 3 and 4, different negative feedback was provided to participants to investigate the effects of various negative feedback on the dissipation of self-deceptive behaviors. Inter-group and intra-group comparison results suggest that the answer group exhibited self-deception under the no-feedback and ambiguous negative feedback in Tests 2, 3, and 4. Under the real negative feedback, self-deception was observed in Test 2 but was notably absent in Tests 3 and 4.These results indicate that while ambiguous negative feedback can effectively reduce self-deception, real negative feedback is more effective in accelerating its decline. These results also indicated that self-deception decayed under the condition of real negative feedback. Under the experimental operation of money reward, the time for self-deception decay was basically the same by inter-group and intra-group comparison measured.

## General discussion

The present study employed a forward-looking paradigm to investigate the effects of repeated negative feedback on the decay of self-deception. The findings from Experiments 1 and 2 demonstrated that the answer group engaged in cheating by accessing the answers and subsequently developed positive beliefs about their performance across all conditions, and indicated that the answer group engaged in self-deception under all conditions in Tests 1 and 2. For Tests 2, 3 and 4, self-deception in the ambiguous negative feedback condition were significantly higher than those in the real negative feedback condition. Additionally, monetary rewards resulted in a convergence of the variability in self-deception between inter-group and intra-group comparisons for Tests 3 and 4. The findings indicate that both ambiguous and real negative feedback can effectively mitigate self-deception, although real negative feedback is more potent in hastening its reduction. Negative feedback fosters the decay of self-deception by diminishing unrealistically positive beliefs. Furthermore, the allure of monetary rewards may predict an increase in instances of cheating behavior, and such rewards also appear to reduce the time required for the decay of self-deception.

### Negative feedback promotes the decay of self-deception

In Experiment 1 and 2, answer hints could induce cheating to establish positive beliefs for estimated scores of answer group for Test 1, so that participants of answer group occurred self-deception behavior with positive beliefs for predicted scores in Test 2. According to the belief adjustment theory (Johnson-Laird et al., 2004), positive beliefs in self-deception can be changed through inconsistent beliefs Self-deception can gradually decay through repeated interventions with negative beliefs of negative feedback (Liu et al., 2019). The results of our research showed self-deception decayed after repeated negative feedback, but the self-deception was not eliminated immediately after the negative feedback. It gradually disappeared over time after repeated feedback interventions under monetary reward. The effectiveness of negative feedback in reducing self-deception may be influenced by the underlying motivation and the timing of its administration. The research results were consistent with belief adjustment theory. When faced with inconsistent information, the original belief is not completely modified, but the intensity is readjusted. The adjustment of beliefs is not a complete overhaul but rather a modification of their intensity. However, self-deception is usually unconscious, so that it is very difficult for individuals to monitor it through meta-cognition or by getting negative feedback from others. Individuals are often reluctant to provide real negative feedback to others, as it can provoke anger, conflict, and psychological distress in them (Audia and Locke, 2003). Anyhow, negative feedback intervention from others or outside world is an important way to decay self-deception.

### Reduction of unrealistically positive beliefs

The results of Experiment 2 demonstrated that predicted scores in the ambiguous negative feedback condition were markedly superior than those in the real negative feedback condition, consistent with the ambiguous condition theory proposed by Sloman et al. (2010). People deceive themselves through the uncertainty of internal reasons and behavior representation (Fernbach et al., 2014). When individuals are uncertain about behavior evaluation in the ambiguous negative feedback, it is easy for them to hold on to positive beliefs of self-deception. When individuals received negative information in the form of real feedback, their predicted scores in Experiment 2 aligned with their actual scores. The results showed that under the real negative feedback condition, self-deception can disappear. Therefore, we could deduce that the existing positive belief in self-deception was an unreal self-positive belief that was still maintained in the face of real negative evidence. Previously, the concept of self-deception has often been confused with other forms of positive bias, such as self-positive bias (Lee et al., 2023; Li and Nie, 2021) and self-serving bias (Von Hippel and Trivers, 2011). But there is a fundamental distinction where positive beliefs are unreal or real positive beliefs through positive bias generated. Only when individuals give up their efforts in failure, but still believe in the unreal positive belief that “failure is the mother of success” can be considered self-deception.

### The influence of monetary reward on the decay of self-deception

In Experiment 1, self-deception completely disappeared for Test 3 in the ambiguous negative feedback, while self-deception still existed in Experiment 2. Only the real negative feedback of Test 3, self-deception disappeared. These results suggest that monetary rewards reduce the time for self-deception decay. In other words, while Chance et al. (2011) suggests that monetary rewards are not essential to enhance individual cheating or self-deception, the allure of such rewards may predict an increase in cheating behavior(Chen et al., 2024), which in turn promotes self-deception. Self-deception, in turn, facilitates cheating by reducing cognitive load and diminishing the specific cues associated with deception (Butterworth et al., 2022). Therefore, this relationship suggests that deception and self-deception are interdependent and mutually reinforcing (Liu et al., 2019). These findings suggest that external motivation could be a contributing factor in self-deception. As Hrgović and Hromatko (2019) argued, self-deception may involve unconscious processes of deception, influenced not only by internal factors but also by external factors.

### Limitations and future study

Drawing on belief adjustment theory and the ambiguous feedback theory of self-deception, we discovered a novel approach to mitigate the decay of self-deception through negative feedback. In comparison with previous studies, this research had achieved some innovations. Firstly, we systematically investigated how the decay of self-deception differs over time under various types of negative feedback, including ambiguous and real feedback conditions. Secondly, this study addresses a gap in the literature, as previous research only examined either ambiguous or concrete feedback. The current study found that the distinction between individuals with real and unreal beliefs accounts for the difference between positive self-deception and other positive beliefs, providing a more in-depth theoretical basis and behavioral evidence for self-deception decay. Finally, our research demonstrated that monetary rewards can be regarded as external motivators influencing the duration of self-deception. This finding paves the way for new avenues of inquiry into the motivations underlying self-deception.

However, our study still had some limitations to be addressed by future research. On one hand, repeated exposure to real feedback led the self-deception levels in the answer group to decrease to those of the control group (Chance et al., 2015; Tang et al., 2018). However, this study found that self-deception could also be reduced with a single instance of real negative feedback. The discrepancy may be due to our use of negative feedback as a within-subject variable. On the other hand, self-deception in response to real negative feedback might have been influenced by prior ambiguous negative feedback. Future research could explore the decay of self-deception by treating negative feedback as a between-subject variable. Additionally, this study examined the effects of different negative feedback on self-deception by incorporating monetary rewards as external motivation. Future studies might consider investigating the decay of self-deception through the lens of monetary rewards or other external incentives. Moreover, given the complex psychological structure of self-deception (Uziel and Cohen, 2020), techniques like ERP or fMRI could be employed to further explore the neural mechanisms underlying self-deception reduction.

## Data Availability

The raw data supporting the conclusions of this article will be made available by the authors, without undue reservation.
